# Retraction: *Ginkgo biloba* Extract Decreases Non-Small Cell Lung Cancer Cell Migration by Downregulating Metastasis-Associated Factor Heat-Shock Protein 27

**DOI:** 10.1371/journal.pone.0335087

**Published:** 2025-10-22

**Authors:** The PLOS One Editors

After this article [1] was published, concerns were raised regarding results presented in Figs 5 and 6.

Specifically:

- There appear to be repetitive areas within the following panels:
  - ◦ Fig 5B NC-siRNA
  - ◦ Fig 6E EGb761
  - ◦ Fig 6E EGb761+LY
- There appear to be similarities between parts of the following panels:
  - ◦ Fig 5A HSP27-siRNA and Fig 5A EGb761
  - ◦ Fig 5B Control, Fig 5B NC-siRNA, and Fig 6E EGb761+LY
  - ◦ Fig 5B HSP27-siRNA and Fig 6E EGb761
  - ◦ Fig 6E Control and Fig 6E EGb761+SB

First author JRT provided some of the underlying microscopy data for some of the panels in Fig 5 as well as repeat data conducted after the time of the original experiments for Fig 5A, but stated that not all the original microscopy data underlying Figs 5 and 6 are available. The underlying data provided did not resolve the above concerns.

In light of the above unresolved concerns that question the reliability and integrity of the reported results, the PLOS One Editors retract this article.

PLL agreed with the retraction. JRT and IWC did not agree with the retraction. JRT and PLL apologize for the issues with the published article. IWC stands by the article’s findings. YHC, SHC, MCY, YJC, JJH, and WHY either did not respond directly or could not be reached.
